# Genome-wide association study for growth traits in Blanco Orejinegro and Romosinuano cattle

**DOI:** 10.1007/s11250-023-03743-9

**Published:** 2023-10-17

**Authors:** Diego H. Bejarano, Rodrigo A. Martínez, Juan F. Rocha

**Affiliations:** Corporación Colombiana de Investigación Agropecuaria -AGROSAVIA. Centro de Investigación Tibaitatá, Km. 14, Mosquera, Cundinamarca Colombia

**Keywords:** Colombian cattle breeds, Birth weight, Postnatal growth, Weaning weight, Single nucleotide polymorphism (SNP), Quantitative trait loci (QTL)

## Abstract

Growth traits are economically important characteristics for the genetic improvement of local cattle breeds. Genome-wide association studies (GWAS) provide valuable information to enhance the understanding on the genetics of complex traits. The aim of this study was to perform a GWAS to identify genomic regions and genes associated to birth weight, weaning weight adjusted for 240 days, 16 months, and 24 months weight in Romosinuano (ROMO) and Blanco Orejinegro (BON) cattle. A single-step genomic-BLUP was implemented using 596 BON and 569 ROMO individuals that were genotyped with an Illumina BovineSNP50 BeadChip. There were 25 regions of interest identified on different chromosomes, with few of them simultaneously associated with two or more growth traits and some were common to both breeds. The gene mapping allowed to find 173 annotations on these regions, from which 49 represent potential candidate genes with known growth-related functions in cattle and other species. Among the regions that were associated with several growth traits, that at 24 – 27 MB of BTA14, has important candidate genes such as LYPLA1, XKR4, TMEM68 and PLAG1. Another region of interest at 0.40–0.77 Mb of BTA23 was identified in both breeds, containing KHDRBS2 as a potential candidate gene influencing body weight. Future studies targeting these regions could provide more knowledge to uncover the genetic architecture underlying growth traits in BON and ROMO cattle. The genomic regions and genes identified in this study could be used to improve the prediction of genetic merit for growth traits in these creole cattle breeds.

## Introduction

Growth traits are commonly used as a selection criterion for the genetic improvement of beef cattle, due to its association with beef production and slaughter age, which have great economic importance for farmers and the food industry (Barwick and Henzell, 2005; Doran et al., 2014). Growth traits regularly used for selection are those measurements of body weight that can be recorded from birth and throughout the life of the animal (Buzanskas et al., 2014). Generally, heritability and genetic correlation coefficients for growth traits are medium to high (Baldi et al., 2010; Gaviolli et al., 2012; Ossa et al., 2014). Therefore, selection based on the genetic merit for these traits and applied over multiple generations has been effective to increase the postnatal growth in most beef cattle breeds (Bennett et al., 2008; MacNeil, 2003; MacNeil et al., 1998). However, the genetic progress of such traits can be accelerated if DNA polymorphisms responsible of the genetic variation in birth weight and postnatal growth are determined and included in the estimation of the animals genetic merit (Dekkers, 2012; Meuwissen et al., 2001; VanRaden, 2020).

In recent years, whole-genome sequencing technologies allowed the identification of a large number of variations in the animals DNA, mainly single nucleotide polymorphisms (SNP) (Zhang et al., 2012). Both the availability of high-throughput genotyping of SNPs, such as high-density micro-arrays (Matukumalli et al., 2009; Van Tassell et al., 2008), and the improvement of statistical methods for genomic analysis (Nicolazzi et al., 2015) contributed to the development of a new methodology for searching candidate genes, known as genome-wide association studies (GWAS) (Hayes and Goddard, 2010; Hirschhorn and Daly, 2005; Zhang et al., 2012). In GWAS, the information of thousands of SNPs distributed uniformly throughout the genome is used, together with the animals’ phenotypes and pedigree information, to perform association analysis and identify genes or regulatory elements involved in the control of traits of economic importance (Chan et al., 2009; Goddard and Hayes, 2009). This methodology has become the method of choice to study the genetic mechanisms that control the expression of complex quantitative traits (MacLeod et al., 2010; Zhang et al., 2012). Imputation of missing genotypes is important to join data from animals genotyped on different SNP panels, which might occur due to the availability of different technologies or for economic reasons (Druet et al., 2010). Procedures to infer or to impute SNPs from high density genotyping chips for animals that are genotyped with lower density chips have been developed (Piccoli et al., 2014).

In recent decades, Colombia has made substantial efforts on the conservation of its animal genetic resources to ensure the adaptability of livestock production systems (González et al., 2020; Jimenez et al., 2021). To promote the use of local cattle breeds in beef farms and to improve their growth traits, a genetic breeding program was set up. Currently, studies are being conducted on the applicability of genomic data to improve the accuracy of the selection process in Colombian creole cattle populations (Burgos-Paz and Martinéz, 2019; Toro et al., 2020), and genome-wide association studies (GWAS) provide valuable information to enhance the understanding on the genetics of complex traits as growth, which could increase selection response to obtain animals with rapid growth at early ages (An et al., 2020; Igoshin et al., 2019; Seabury et al., 2017; Smith et al., 2019).

The objective of the present study was to perform a GWAS to identify genomic regions and genes that might affect birth weight, weaning weight, 16-month weight, and 24-month weight in the Colombian creole cattle breeds Romosinuano (ROMO) and Blanco Orejinegro (BON).

### Materials and methods

#### Animals and phenotypes

A genealogical database of 7078 ROMO and 8255 BON individuals was used, including 4063 ROMO and 3922 BON with productive records for one or more of the following traits: birth weight (BW), weaning weight adjusted for 240 days of age (WW), weight at 16 months (16mW), and weight at 24 months (24mW). Both genealogy and phenotypes were retrieved from the databases of the Colombian national animal germplasm bank. ROMO individuals were kept in the Turipaná research center located in the valley of the Sinú river in Cereté, Córdoba, Colombia (Caribbean region), with an average temperature of 27.5 °C, relative humidity of 83%, and an annual precipitation of 1200 mm, distributed in a low precipitation season from December to March and another period of high precipitation from April to November. ROMO cattle were fed with a mixture of *Dichanthium aristatum* (angleton grass), *Megathyrsus maximus* (guinea grass) and *Cynodon* spp. (stargrass). BON individuals were kept in the El Nus research center located in San Roque, Antioquia, Colombia (Andean region), with an annual average temperatures between 18 and 24 °C and a bimodal rainfall regime. BON cattle were fed with a mixture of *Brachiaria decumbens* (signal grass), *Brachiaria brizantha* (palisade grass), and *Cynodon plectostachyus* (giant star grass).

#### Genotyping and quality control

Blood (germplasm bank *in vivo*) and semen (germplasm bank *in vitro*) samples were collected, and DNA extraction was carried out in the molecular genetics’ laboratory of AGROSAVIA, employing a commercial kit (MoBio Laboratories Inc., CA, USA). The concentration (≥ 50 ng/ml) and integrity (ratio = 1.8) of the DNA was evaluated in a NanoDrop 2000®. A total of 596 BON and 569 ROMO cattle were genotyped for 52784 polymorphisms. This information was obtained from two different SNP panels of high density, the BovineSNP50K_v2 (Illumina Inc., 2016), which includes 54609 SNPs and the BeadChip IlluminaSNP7K that includes 6909 SNP (Illumina Inc. 2016). The BovineSNP50K_v2 (50 K) was used for genotyping 866 animals, which had both phenotypes and genealogical information, while the IlluminaSNP7K (7 K) was used for genotyping 299 animals, mainly offspring with phenotypic information.

Quality control procedures were carried out with PLINK software v1.9 (Purcell et al., 2007) as follows. First, animals with > 10% missing genotypes or an SNP Mendelian error rate > 2% were removed. Then, SNPs with a call rate < 90%, a minor allele frequency < 1%, and a Hardy–Weinberg equilibrium test *P* value < 0.01 were removed. After QC procedures, 40,555 SNPs in BON and 40,421 SNPs in ROMO were available for GWAS.

#### Imputation of genotypes

Because two genotyping panels of different SNP density were employed, there were large fractions of missing genotypes for individuals genotyped with the low-density chip (7 K) after combining both data sets. Therefore, genotypes of SNPs included in the high density chip (BovineSNP50_v2) were imputed for all animals genotyped with the low-density panel (IlluminaSNP7K), using the FImpute software (Sargolzaei et al., 2014).

#### Population structure

A principal component analysis (PCA) was used to examine the genetic structure of the two populations. The PCA was performed using a genomic identity-by-state (IBS) relationship matrix.

#### Model and computing

A single-step genomic association study (ssGWAS) was performed, which is an alternative approach for association analyses proposed by Wang et al. (2012), based on the method of single step genomic-BLUP (ssBLUP) (Aguilar et al., 2010; Misztal et al., 2013). The model considered additive genetic relationships between the individuals, combining pedigree and genomic information into the H matrix (Aguilar et al., 2010; Legarra et al., 2009). The inverse of the matrix H is constructed by combining the inverse of the SNP-derived genomic matrix (G) and the pedigree numerator relationship matrix (A), as follows:

$$ {H}^{-1}={A}^{-1}+\left[\begin{array}{cc}0& 0\\ {}0& {G}^{-1}-{A}_{22}^{-1}\end{array}\right] $$

where A^−1^_22_ is the inverse of the numerator relationship matrix for the genotyped individuals and *G* is a matrix of genomic relationships. The matrix G was constructed weighting each marker contribution by its expected variance, according to the methodology described by VanRaden (2008), with G = ZDZ', where *D* is a diagonal matrix with elements containing the inverse of the expected marker variance ${D}_{ii}=\frac{1}{m[{2p}_{i}\left(1-{p}_{i}\right)]}$ and *Z* is the marker incidence matrix containing genotypes (0, 1 or 2) corrected by allele frequency (VanRaden, 2008).

The H^−1^ matrix was replaced within the mixed model equation, and an animal model was implemented for each breed and trait including the fixed effects of sex, year, and season birth (rainy and dry) and the random effects of animal direct additive genetic, maternal additive genetic, maternal permanent environment (only for BW and WW), and residual variance, which were all set in the following mixed linear model:

$$Y=1\mu +X\beta +{Z}_{a}a+e$$

where *Y* is the vector of observations for the respective trait evaluated, 1 is all-ones vector, *µ* is the overall mean for the phenotypic records, *β* is a vector of fixed effects, *X* is the corresponding incidence matrix for the fixed effects, $a$ is the vector of direct additive genetic effects assuming $a$~*N(0, Hσ*^*2*^_*a*_), where *H* represents the additive relationship matrix that combines pedigree and genomic information, ${Z}_{a}$ is the incidence matrix for the animal additive genetic random effect and *e* is a vector of residual random effects with ~ *N(0, Iσ*^*2*^_*e*_*)*. The variance–covariance structure of the additive genetic effects was Var(animal) = ${H\sigma }_{a}^{2}$, where* H* is a matrix of additive genomic relationships among individuals built from SNPs data combined with pedigree data and ${\sigma }_{a}^{2}$ is the additive genetic variance.

Variance components were estimated by REML based on all the individuals in the pedigree (Thompson and Mäntysaari, 1999). All analyses for REML, BLUP, and ssGWAS were performed using the BLUPF90 software (Aguilar et al., 2011; Misztal et al., 2002). PREGSF90 and POSTGSF90 packages were used to perform the ssGWAS for each trait. Candidate regions associated with BW, WW, 16mW, and 24mW in each breed were identified based on the amount of genetic variance explained by windows of 4 adjacent SNPs evaluated across the entire bovine genome. After obtaining the genomic estimated breeding values (GEBVs), the SNP effects were estimated as:

$$ \hat{u}=D{Z}^{\prime }{\left[ ZDZ^\prime \right]}^{-1}{\hat{a}}_g $$

where *û* is the vector of SNP marker effects, *D* is a diagonal matrix of weights of SNPs, *Z* is a matrix related to the genotypes of each locus for each individual, and ${\widehat{a}}_{g}$ is the vector of GEBVs for animals with genotype (Wang et al., 2012). The percentage of genetic variance explained by windows of nth adjacent SNPs was calculated as:

$$\frac{Var ({a}_{i})}{{a}_{a}^{2}} x 100\%=\frac{Var({\sum }_{j=1}^{4} {Z}_{j }{\hat{u} }_{j })}{{\sigma }_{a}^{2}}\times 100\%$$

where *ai* is the genetic value of the *i*-th genomic region that consists of 4 adjacent SNPs, ${\sigma }_{a}^{2}$ is the total genetic variance, ${Z}_{j}$ is vector of gene content of the* j*-th SNP for all individuals, and ${\hat{u} }_{j}$ is marker effect of the *j*-th SNP within the *i*-th region. GWAS results were plotted with qqman package included in R to build Manhattan plots (Turner, 2014).

#### Gene mapping and in silico functional analyses

We selected the windows explaining the largest amount of genetic variance (≥ 0.15%) to find the surrounding genes within 500 kb. Based on the starting and ending coordinates of the windows, gene annotations were obtained using the genome databanks National Center for Biotechnology Information (NCBI, 2019) and Ensembl Genome Browser (Ensembl, 2019). The functional analysis of the mapped genes was conducted via the website of UniProt (2019) and GeneCards (2019) to verify the biological function of these genes and their possible relation to growth traits and when no information was available for the *Bos taurus* genes, annotations from human, rat, or mouse orthologs were used to proceed with the in silico functional analyses. AnimalQTLdb (2019) was accessed to verify the previous QTL reported for growth traits in the regions of interest.

## Results

**Fig. 1:**
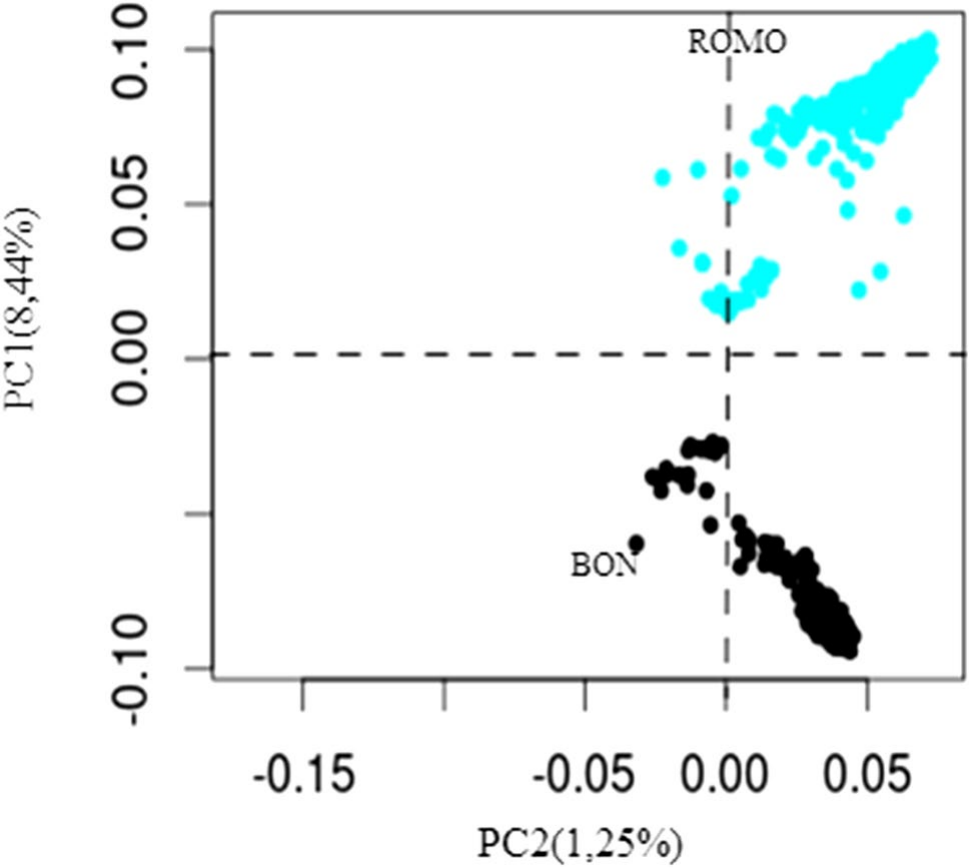
PCA derived from the genomic identity-by-state (IBS) relationship matrix

The results of the PCA show that the first principal component (PC) explains 8.44% of the total variation and clearly separates the BON breed (black) from the ROMO breed (blue), while the second PC explains only the 1.25% of the variance (Fig. 1). The spatial distribution of the animals assigned to each breed indicates that they are genetically close populations, which have a definite genetic structure, with little dispersion within each breed; this suggests that stratification within the evaluated populations is low.

In this study, the windows of 4 adjacent SNPs that accounted for more than 0.15% of the genetic variance were used to search for putative candidate genes (PCG), which are represented in Table 1 and Table 2. A total of 49 genomic regions were identified through ssGWAS, which explained more than 0.15% of the genetic variance for one or more of the four growth traits analyzed in both BON and ROMO cattle.

**Table 1:** Genomic regions associated with BW, WW, 16mW, and 24mW in ROMO cattle

QTL position		Variance explained (%) SNP window^a^				PCG^b^
BTA	QTL window (Mb)	BW	WW	16mW	24mW	
2	04.13–04.24				0.16	MSTN
2	108.79–108.87				0.17	IGFBP2, IGFBP5
2	78.30–78.41			0.23		-^c^
3	87.81–87.90		0.16			MYSM1, TACSTD2, OMA1
4	20.40–20.51		0.3	0.25		TMEM106B
4	39.12–39.28		0.15	0.17		HGF, CACNA2D1
4	91.13–91.23	0.28				LEP, GRM8
5	02.10–02.19	0.15				TRHDE
5	06.46–06.55	0.17				CSRP2
5	10.51–10.66	0.17				Myf5, Myf6
5	18.00–18.08		0.41	0.43	0.45	KITLG, TMTC3
9	24.94–25.06				0.27	-
9	86.79–86.88			0.18		-
11	02.11–02.20				0.16	ENSBTAG00000011553
11	59.31–59.42		0.24	0.17		-
13	19.94–20.01				0.16	NPR1
13	22.06–22.21			0.15		PNR1
13	28.09–28.19			0.17		MCM10, UCMA
13	28.10–28.18				0.27	MCM10, UCMA
14	24.52–24.64		0.58	0.52	0.25	XKR4, TMEM68, PLAG1
15	10.51–10.70		0.15			-
19	14.92–15.06			0.2		Gas2L2
19	15.00–15.11				0.24	Gas2L2
23	00.40–00.76		0.15	0.24	0.21	KHDRBS2 (SLM1)
23	14.17–14.25			0.15	0.17	-
26	08.22–08.36	0.15				PRKG1
# Regions of interest^d^		5	8	12	11	

^a^Window that consists of 4 continuous SNPs

^b^Positional and functional/putative candidate gene

^c^No PCG associated with the trait

^d^Regions that accounted for ≥ 0.15% of the genetic variance

**Table 2:** Genomic regions associated with BW, WW, 16mW, and 24mW evaluated in BON cattle

QTL position		Variance explained (%) SNP window^a^				PCG^b^
BTA	QTL window (Mb)	BW	WW	16mW	24mW	
3	79.48–79.58	0.19				LEPR
4	92.05–92.18			0.18		LEP, GRM8, PAX4
5	40.51–40.58				0.21	CNTN1
6	67.17–67.24		0.2			GABRA4, GABRA1
6	82.48–82.61	0.15				-^c^
11	20.78–20.90				0.15	-
11	70.83–71.09			0.15		SPDYA, PPP1CB, FOSL2
14	08.11–08.24	0.16				ZFAT, TG
14	11.74–11.85			0.16		TG
14	19.38–19.49	0.24				-
14	21.34–21.45	0.16				PRKDC, MCM4, SNAI2
14	21.66–21.87	0.22				-
14	23.82–23.95	0.15				SOX17, LYPLA1
14	26.45–26.54	0.18				-
14	26.71–26.84	0.18				PLAG1
14	26.87–26.95	0.26				PLAG1
14	27.23–27.32	0.36	0.22	0.17		PLAG1
14	43.90–44.03	0.2				PKIA
16	20.05–20.13				0.26	-
20	34.31–34.39			0.19		GHR
20	44.45–44.55				0.24	-
23	00.40–00.76		0.22	0.42		KHDRBS2 (SLM1)
25	03.62–03.70				0.16	MGRN1
25	40.41–40.55		0.17			-
27	21.47–21.58				0.16	SGCZ
# Regions of interest^d^		12	4	6	6	

^a^Window that consists of 4 continuous SNPs

^b^Positional and functional/putative candidate gene

^c^No PCG associated with the trait

^d^Regions that accounted for ≥ 0.15% of the genetic variance

**Fig. 2:**
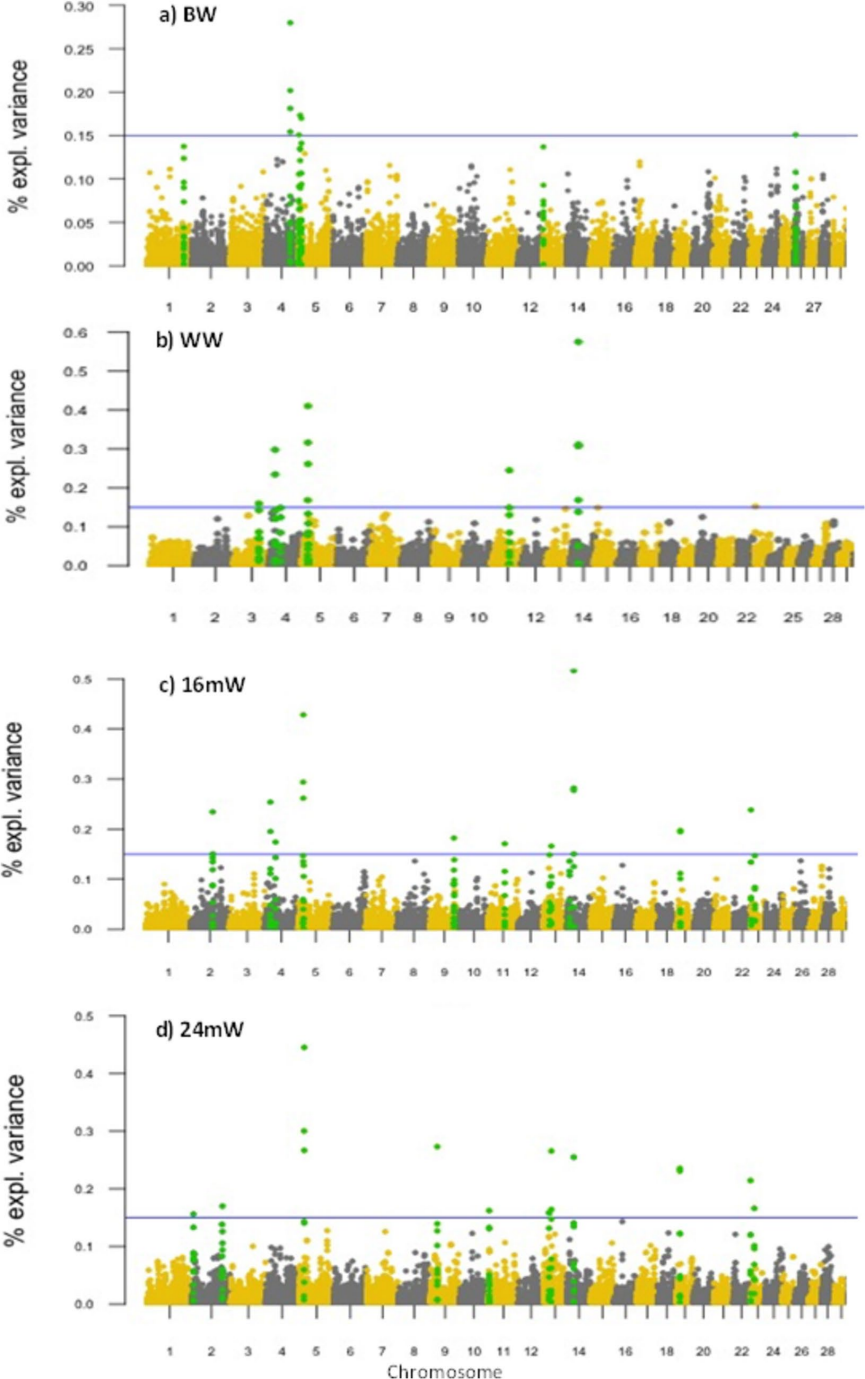
Manhattan plot of the genome-wide association study for BW (**a**), WW (**b**), 16mW (**c**), and 24mW (**d**) in ROMO breed. The *X*-axis represents the chromosomes (29 autosomal), and the *Y*-axis shows the proportion of genetic variance explained by windows of 4 adjacent SNPs for each growth trait in ROMO. The regions that explain a higher proportion of variance are highlighted in green

**Fig. 3:**
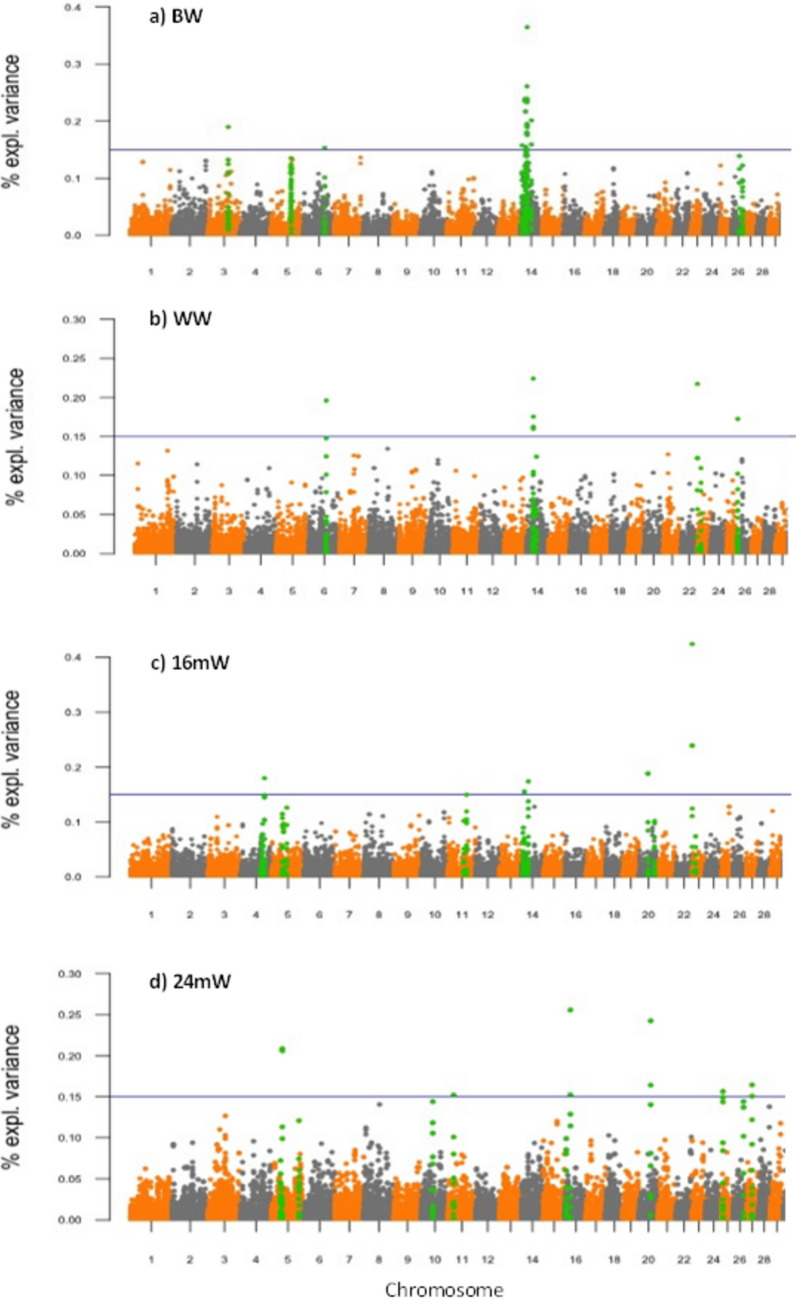
Manhattan plot of the genome-wide association study for BW (**a**), WW (**b**), 16mW (**c**), and 24mW (**d**) in BON breed. The *X*-axis represents the chromosomes (29 autosomal), and the *Y*-axis shows the proportion of genetic variance explained by windows of 4 adjacent SNPs for each growth traits in BON. The regions that explain a higher proportion of variance are highlighted in green

The results of the ssGWAS are shown in Fig. 2 and in Fig. 3 (Manhattan plots), where the proportion of genetic variance (%gVar) explained by windows of 4 adjacent SNPs is shown graphically for BW, WW, 16mW, and 24mW in ROMO and BON, respectively. In the Manhattan plots, the chromosomes are differentiated by colors, and the SNPs are represented by individual points, with a spatial distribution that depends on the %gVar explained by the 4-adjacent SNPs that make up each window (*Y*-axis) and the position that SNP occupies in pairs of bases (bp) within each chromosome (*X*-axis).

The details of the regions of interest identified in the ssGWAS are presented in Table 1 and Table 2. Five genomic regions that explain more than 0.15% of the gVar for BW in ROMO were located on BTA4, BTA5 and BTA26 (Fig. 2a). Most of the 12 regions associated with BW in BON are located on BTA14 and some on BTA3 and BTA6 (Fig. 3a). For WW, eight regions were found associated in ROMO, on BTA3, BTA4, BTA5, BTA11, BTA14, BTA15, and BTA23 (Fig. 2b), and four regions in BON, on BTA6, BTA14, BTA23, and BTA25 (Fig. 3b). Moreover, 12 regions across nine different chromosomes (BTA2, BTA4, BTA5, BTA9, BTA11, BTA13, BTA14, BTA19, and BTA23) were associated with 16mW in ROMO (Fig. 2c), and 6 regions were associated with this trait in BON, on BTA4, BTA11, BTA14, BTA20, and BTA23 (Fig. 3c). Finally, the ssGWAS showed 11 regions associated with 24mW in ROMO, on BTA2, BTA5, BTA9, BTA11, BTA13, BTA14, BTA19, and BTA23 (Fig. 2d), and 6 associated regions for this trait in BON on BTA5, BTA11, BTA16, BTA20, BTA25, and BTA27 (Fig. 3d).

Some genomic regions were simultaneously associated with two or more growth traits and in some cases were common to both breeds. Chromosomal regions with simultaneous effect for several traits were more frequent in ROMO (Table 1), for example, two regions in BTA4 were simultaneously associated with WW and 16mW. The first region (20.39–20.51 Mb) explains the 0.3% and 0.25% of the gVar of these two characteristics, respectively, and the second region (39.12–39.29 Mb) explains the 0.15% of the gVar of WW and the 0.17% of the gVar of 16mW. Also, a region on BTA5 (17.99–18.08 MB) had common effect on WW (0.43% gVar), 16mW (0.45% gVar), and 24mW (0.41% gVar). Likewise, a region on BTA11 (59.31–59.43 Mb) showed a common effect on WW (0.24% gVar) and 16mW (0.17% gVar), while that at 28.09–28.19 Mb of BTA13 explained 0.17% gVar of 16mW and 0.27% gVar of 24mW.

Multiple regions associated with several growth traits were found in chromosome 14. Interestingly, eight regions associated with BW in BON were very close to each other at 19.3–27.3 Mb of BTA14. Another region at 27.23–27.33 Mb of BTA14 in BON showed an important effect on BW (0.37% gVar), WW (0.22% gVar), 16mW (0.15% gVar), and 24mW (0.1% gVar). Close to this location on BTA14 (24.52–24.65 Mb), but in ROMO, another region was highly associated with WW (0.57% gVar), 16mW (0.52% gVar), and 24mW (0.25% GVar) (Fig. 3).

A particularly interesting region at 0.44–0.77 MB of BTA23 was associated with the genetic variation of several growth traits in both breeds. In ROMO, this region was associated with WW (0.15% gVar), 16mW (0.24 gVar), and 24mW (0.21% gVar) (Fig. 2). In BON, the same region showed a significant effect on WW (0.22% gVar), 16mW (0.42% gVar), and 24mW (0.10% gVar) (Fig. 3). Additionally, the region at 14.17–14.25 MB of BTA23 was simultaneously associated with 16mW (0.15% gVar) and 24mW (0.16% gVar) but only in ROMO cattle.

After performing the gene mapping for all regions of interest identified in both breeds (Fig. 2 and Fig. 3), a total of 173 annotations were found, some of them with known functions, including 53 possible positional and functional candidate genes that have a function directly or indirectly related to the regulation of growth in cattle and other species (Tables 1 and 2).

## Discussion

These results are consistent with other genome-wide association studies, which have identified a common region on BTA14, between 20 and 30 Mb, containing SNPs associated with productive traits such as age at puberty in males and females, serum levels of insulin-like growth factor type I (IGF-I), weight at different ages, hip height, and deposition fat in Brahman cattle (*B. indicus*) and composite tropical breeds (Fortes et al., 2012; Hawken et al., 2012). This region on BTA14 was found to be associated with fat deposition phenotypes, evaluated after slaughter, in studies that included *B. taurus* cattle, *B. indicus*, composite tropical breeds, and cross-bred animals (*B. indicus/B. taurus*) (Bolormaa et al., 2011a, 2011b; Porto Neto et al., 2012). Other studies have found this same BTA14 region associated with height, size, and weight at different ages in populations of dairy cattle *B. taurus* (Karim et al., 2011; Littlejohn et al., 2011) and in a population of Japanese black cattle (Nishimura et al., 2012). Also, Lindholm-Perry et al. (2012a, 2012b) and Snelling et al. (2011) have reported that SNPs in this region were associated with other important traits such as food intake, residual feed intake, and the average daily gain. This suggests that one or more mutations located between 20 to 30 MB in the BTA14 could have pleiotropic effects on different productive traits in cattle (Fortes et al., 2013).

Most of the potential candidate genes found in BTA14 such as PLAG1 (14:25.00–25.05 MB), SNAI2 (14:21.57–21.58 Mb), SOX17 (14:23.88–23.89 Mb), and LYPLA1 (14:23.65–23.67) are involved in replication processes, differentiation, and cell function. Some polymorphisms in PLAG1 have been linked to growth, development, and carcass traits in different cattle breeds, suggesting its possible pleiotropic effect (An et al., 2019; Fortes et al., 2013; Hoshiba et al., 2013; Karim et al., 2011; Littlejohn et al., 2011; Zhang et al., 2019). SNAI2 is involved in various developmental and physiological mechanisms (Hemavathy et al., 2000), like the maturation of osteoblasts (Piva et al., 2011). SOX17 participates in endoderm and vascular development (Kanai-Azuma et al., 2002; Matsui et al., 2006) and plays an important role in fetal hematopoiesis (Nakajima-Takagi et al., 2013). And finally, LYPLA1 could influence appetite and weight gain in livestock (Shanado et al., 2004) and the average daily feed intake in cattle (Lindholm-Perry et al. 2012a).

The region at 24.5–24.6 Mb of BTA14 was associated with three growth traits in ROMO cattle. Some important genes such as XKR4 (14:24.29–24.62 MB) and TMEM68 (14:24.71–24.75 MB) have been mapped. Previous studies identified several SNPs in XKR4 associated with rump fat thickness in Australian and composite cattle (Bolormaa et al., 2011b; Porto Neto et al., 2012), suggesting a likely relationship of this gene with metabolism and fat deposition in livestock. Also, An et al. (2019) reported the XKR4 gene as a candidate gene associated with body measurement traits in Chinese Wagyu beef cattle. Lindholm-Perry et al. (2012a, 2012b) identified five SNPs near TMEM68 and XKR4 genes that were strongly associated with variation in feed intake and weight gain.

Interesting regions associated with several growth traits in both breeds were observed on BTA23. For instance, that at 23: 0.40–0.77 MB was associated with WW, 16mW, and 24mW, in both ROMO and BON cattle. This suggests that there is one or more conserved genes within or near this region that affect the expression of characteristics associated with growth in Colombian cattle breeds (Figs. 2 and 3). Moreover, the candidate gene SLM1 at 23: 0.18–0.86 Mb) encodes for a protein highly expressed in cardiac and skeletal muscle (Brown et al., 1999). It has been shown that SLM1 regulates events mediated by integrin, including the migration and spread of myoblasts, and hypertrophic signaling in the myocardium (Robinson et al., 2003). Previous studies in BON and ROMO (Martinez et al., 2014) found several polymorphisms in BTA19 and BTA23 associated with BW. Most of the regions identified in the present study that were simultaneously associated with several growth traits or that were common for both breeds were identified in these two chromosomes, which might indicate their importance to explain the genetic and phenotypic variation of growth traits in Colombian cattle breeds.

A genomic region at 79.48 Mb of BTA3 was associated with BW in BON (Fig. 3a), containing two candidate genes, the leptin receptor (LEPR) and leptin receptor overlapping transcript (LEPROT). These genes play an important role in regulating body energy homeostasis and metabolism (de Luis Roman et al., 2006; Guo et al., 2008) and are involved in the control of the growth hormone (Belgareh-Touzé et al., 2002), which may constitute a molecular link between nutritional signals and the actions of GH in body growth and metabolism (Belgareh-Touzé et al., 2002; Touvier et al., 2009; Wu et al., 2013). On the other hand, the region at 87.8 Mb of BTA3 associated with WW in ROMO (Fig. 2b) has potential candidate genes such as TACSTD2 and OMA1. These genes are involved in postnatal growth and childhood fat mass in humans (Groom et al., 2012), and in lipid and glucose metabolism in both humans and mice, and have been linked to the phenotype of obesity (Head et al., 2009; Quirós et al., 2012). Some SNPs found near this region on BTA3 by Bolormaa et al. (2011a) were significantly associated with residual feed intake, average daily gain and metabolic body weight in beef cattle. In the GWAS carried out by Londoño-Gil et al. (2021) in BON cattle, two regions on BTA1 and BTA3 had an effect on WW, daily weight gain between birth and weaning (DWG), yearling weight (YW), time to reach 120 kg of live weight (T120), and time to reach 60% of adult weight (T60%). Like our study, they found a region on BTA3 associated with BW and WW. Differences with the associations found in our study could be due to the methods of analysis implemented and the reference populations used.

The region at 39.1 Mb of BTA4 associated with WW and 16mW in ROMO contains the candidate gene HGF, which is one of the multifunctional cellular factors that regulates cell proliferation, cell motility, and morphogenesis in mammals (Asami et al., 1991; Schmidt et al., 1995). Cai et al. (2013) also found SNPs in HGF significantly associated with growth characteristics in Chinese cattle. Additionally, Yuan and Xu (2011) observed SNPs on the candidate gene CACNA2D1 (4:38.33–38.86 Mb), which were associated with carcass and meat quality traits in cattle. On the other hand, two regions on BTA4 at 91.1 Mb and 92.0 Mb associated with BW in ROMO and with 16mW in BON, respectively, are located near the leptin gene (LEP), which is involved in the regulation of energy metabolism affecting body weight, food intake, energy expenditure, and reproduction (Delavaud et al., 2002; Garcia et al., 2002; Lord et al., 1998; Woods, 1998).

The region at 17.9 Mb of BTA5 associated with WW, 16mW, and 24mW in ROMO contains KITLG and TMTC3, which are involved in regulation of growth of oocytes in cattle (Cho et al., 2008) and the development of myofibroblasts in the pulmonary alveoli and in the bronchial smooth muscle cells (Yun and Vu, 2012). Additionally, another region at 10.5 Mb of BTA5 had a significant effect on BW in ROMO (Fig. 2a) and is located close to the myogenic factors MYF5 and MYF6, which belong to a gene family of myogenic determination factors (MyoD) (Braun et al., 1990, 1989). The number of muscle fibers (myocytes) at birth appears to determine the maximal lean meat growth capacity in pigs and in cattle (Handel and Stickland, 1988, 1987). Mammalian myofiber formation is a strictly embryonic process, regulated by the MyoD gene family. Myf-5 and MyoD1 are expressed during proliferation of myoblasts, myogenin is expressed during terminal differentiation, and myf-6 is mainly expressed during postnatal life (Olson, 1990; Rudnicki and Jaenisch, 1995; Weintraub et al., 1991). Several studies have reported QTL and SNPs in MYF5 associated with variation in skeletal muscle tissue development in pigs and cattle (Li et al., 2003; Robakowska-Hyzorek et al., 2010; Sarti et al., 2014). Therefore, MYF5 is being considered as a potential candidate gene for growth traits.

The UCMA gene, located in a region of BTA13 (28.09 Mb) associated with 16mW and 24mW in ROMO, is involved in the negative control of osteogenic differentiation of osteochondrogenic precursor cells in peripheral zones of fetal cartilage and at the cartilage-bone interface (Surmann-Schmitt et al., 2008; Tagariello et al., 2008). Another region at 19.93 Mb of BTA13 associated with 24mW in ROMO (Fig. 2d) contains PNR1, which regulates diverse biological processes (Bielenberg et al., 2006), acting as coreceptor for some growth factors, such as vascular endothelial growth factor (Gu et al., 2003), hepatocyte growth factor (Sulpice et al., 2008), and platelet-derived growth factor (Banerjee et al., 2006), suggesting that PNR1 is a junction protein to growth factors that is involved in the regulation of growth and cell proliferation in different tissues (Evans et al., 2011).

This study showed 49 DNA regions distributed on chromosomes 2, 3, 4, 5, 6, 9, 11, 13, 14, 15, 16, 19, 20, 23, 25, 26, and 27 that were associated with growth traits in BON and ROMO cattle. These regions explained a high proportion of genetic variance for these traits (> 0.15%) and contain 50 potential candidate genes with known functions related to biological processes of growth in cattle and other animal species. Some of these genomic regions were simultaneously associated with two or more growth traits and in some cases were common to both breeds (on BTA14 and BTA23). Future studies targeting these areas could provide more knowledge to uncover the genetic architecture underlying growth traits in BON an ROMO creole cattle. The genomic regions and genes identified in this study could be used to improve the prediction of genetic merit for growth traits in local cattle breeds, where there is a limitation of performance records and genealogical data.

## Data Availability

Data supporting the conclusions of this article are included within the article. The datasets used and/or analyzed in our study are available from the corresponding author upon reasonable request.
